# Larval pesticide exposure impacts monarch butterfly performance

**DOI:** 10.1038/s41598-020-71211-7

**Published:** 2020-09-02

**Authors:** Paola Olaya-Arenas, Kayleigh Hauri, Michael E. Scharf, Ian Kaplan

**Affiliations:** Department of Entomology, Purdue University, West Lafayette, IN 47907 USA

**Keywords:** Zoology, Ecology, Environmental sciences, Natural hazards

## Abstract

The long-term decline of monarch butterflies has been attributed to loss of their milkweed (*Asclepias* sp.) host-plants after the introduction of herbicide-tolerant crops. However, recent studies report pesticide residues on milkweed leaves that could act as a contributing factor when ingested as part of their larval diet. In this study, we exposed monarch larvae to six pesticides (insecticide: clothianidin; herbicides: atrazine, *S*-metolachlor; fungicides: azoxystrobin, pyraclostrobin, trifloxystrobin) on their primary host-plant, *A. syriaca*. Each was tested at mean and maximum levels reported from published analyses of milkweeds bordering cropland and thus represent field-relevant concentrations. Monarch lethal and sub-lethal responses were tracked over their complete development, from early instar larvae to adult death. Overall, we found no impact of any pesticide on immature development time and relatively weak effects on larval herbivory or survival to adulthood. Comparatively stronger effects were detected for adult performance; namely, a 12.5% reduction in wing length in response to the fungicides azoxystrobin and trifloxystrobin. These data collectively suggest that monarch responses to host-plant pesticides are largely sublethal and more pronounced in the adult stage, despite exposure only as larvae. This outcome has important implications for risk assessment and the migratory success of monarchs in North America.

## Introduction

Agricultural intensification has reduced the abundance and diversity of pollinators such as bees and butterflies in many regions around the world^1–3^. While this reduction is partly due to the indirect effect of habitat loss (i.e., large fields with frequent herbicide inputs have fewer floral resources), intensification can also have direct effects by exposing individuals to pesticide residues that increase mortality or impair development. This is particularly the case for insects developing on flowering plants growing along crop field edges, where pesticide drift or runoff is unavoidable. Several studies now confirm the presence of non-target pesticides in diets of pollinators foraging on non-crop, wild plants^4–9^. Much of these efforts focus on neonicotinoid insecticides due to their rapid adoption by farmers since the 1990s^10^, resulting in a dramatically higher toxic load for pollinators in agricultural landscapes^11,12^.

As a correlative pattern, the abundance of many butterflies has declined with the rise in neonicotinoid use over time^13–15^, but experimental studies testing the impacts of pesticides on non-target butterflies are, in general, limited^16^. For example, a systematic review of four butterfly families—Lycaenidae, Nymphalidae, Hesperiidae, Papilionidae—revealed few published data evaluating the influence of non-target insecticides^17^. In addition, the limited cases overwhelmingly center on insecticides, with comparatively few incorporating fungicides or herbicides. This research gap is noteworthy since some experiments show negative effects of herbicides on Lepidoptera performance, including reduced survival or wing size^18–20^, adult emergence^21^ and sex-pheromone production^22^. Similar studies, mainly on commercial (*Bombyx mori* L.) and pest (*Spodoptera litura* F.) lepidopteran species, have found that fungicides can also reduce larval survival, pupal weight or adult longevity^23,24^. Moreover, fungicides synergize the action of insecticides by inhibiting cytochrome P450 enzymes in honey bees^25–28^. Comparable work in fruit flies revealed that otherwise nontoxic herbicides greatly increase the lethality of insecticides when applied together^29^. Thus, the lack of data on non-insecticidal chemistries, either alone or in combination, prevents a broader assessment of pesticide toxicity for non-target Lepidoptera inhabiting agroecosystems.

A secondary challenge in disentangling the role of pesticides in butterfly population ecology is that chemicals can act on either the larval or adult stage. Although it is assumed that adults may encounter spray drift during flight or residues when contacting pesticide-contaminated surfaces or foods (nectar; e.g.,^30^), less attention is paid to larval host-plants, which could form the bulk of exposure over their full lifecycle^31–33^. Little is known about pesticide concentrations on non-crop plant leaves and how these translate to changes in caterpillar development. Notably, dietary exposure as a larva can have delayed, sublethal effects on adult butterflies. The survival of cabbage butterfly, *Pieris brassicae*, larvae was unaffected by wide variation (up to 200 ppb) of the neonicotinoid imidacloprid in their diet, but even the lowest dose (1 ppb) reduced adult wing size^34^.

The monarch butterfly, *Danaus plexippus* (Lepidoptera: Nymphalidae), is an iconic species whose population is declining in North America. Numerous causal factors have been implicated in their decline^35–37^, with disappearance of its larval host-plant, milkweed (*Asclepias* sp.), due to the introduction of the herbicide glyphosate being the leading hypothesis^38–43^. Some have noted, however, that the decline temporally coincides with the introduction and growing popularity of neonicotinoids and thus speculate that insecticides or other agrochemicals could act as a contributing factor. To date, only a few studies have experimentally assessed the impact of agricultural pesticides on monarch larvae, providing some pieces of the puzzle. Yet, these studies suffer from methodological limitations that prevent us from extrapolating to the broader question of monarch decline. All used ornamental milkweeds that are planted in home gardens such as tropical milkweed *A. curassavica*^44,45^ or swamp milkweed *A. incarnata*^46,47^, rather than the common milkweed *A. syriaca*, which is the most abundant and widely used larval host-plant for eastern migratory monarchs^48,49^. This discrepancy is potentially important since milkweed species differ dramatically in defensive traits affecting host-plant suitability^50^ (e.g., latex, trichomes); for instance, tropical and swamp milkweeds are notable for having extremely high and low levels of cardenolides, respectively^51^. Similarly, experiments mostly employ brief ‘pulsed’ exposure periods with larvae consuming a known insecticide dose over a 24–48 h period^45,46^. This approach is useful in defining acute toxicity and calculating LD_50_ values but does not accurately portray natural exposure where larvae consume trace amounts over their entire developmental period. Additionally, all prior studies investigated insecticides; none have tested other pesticide types, e.g., fungicides, herbicides.

Here, we evaluated the effects of six pesticides—the insecticide clothianidin; the herbicides atrazine and *S*-metolachlor; the fungicides azoxystrobin, pyraclostrobin and trifloxystrobin—and their combination on monarch development. These were among the most commonly detected chemicals on *A. syriaca* leaves in a 2-year field survey of milkweeds bordering cropland^52^. Because we know their specific concentrations, we tested each at mean and maximum levels; thus, all data represent field-realistic pesticide types and doses. Importantly, we exposed monarchs to these treatments on *A. syriaca* leaves over their complete larval period, measuring both immediate (i.e., larval mortality) and delayed responses (i.e., changes in adult size and longevity). These data are ultimately used in risk assessment to estimate the likelihood of lethal or nonlethal effects and their potential to harm monarchs in the wild.

## Methods

### Plants and insects

All experiments were performed using the common milkweed, *A. syriaca*. Seeds (obtained from Prairie Moon Nursery, Winona, MN) were surface sterilized by soaking in a 5% bleach solution, rinsed with distilled water, and the seed coat was nicked with a razor. Seeds were then germinated on moist filter paper in a parafilm-sealed petri dish, covered with aluminum foil and stored at 4 °C for 1–2 weeks, after which they were moved to a growth chamber at 28 °C for 3–4 days. Seedlings were placed in propagation trays filled with SunGro horticulture germination mix with Osmocote 14-14-14 (ICL Specialty Fertilizers), before being moved to a greenhouse and transplanted into 366 mL pots filled with SunGro professional growing mix with two teaspoons of time-released NPK fertilizer (Scotts Osmocote Classic). Biocontrol agents (Koppert Biological Systems)—lacewing (*Chrysoperla carnea*) larvae, predaceous mites (*Amblyseius cucumeris*), entomopathogenic nematodes (*Steinernema feltiae*)—were released on plants regularly to control a diversity of milkweed pests (e.g., thrips, aphids, fungus gnats) without using pesticides.

Monarch eggs were the offspring of wild-caught butterflies from the eastern migratory population. Gravid females were either obtained locally in Indiana or from individuals collected in Georgia at Emory University, provided by Jacobus de Roode.

### Pesticide treatments

We used a larval exposure bioassay using six of the most commonly detected pesticides from our recent field survey^52^. For each, we tested the mean and maximum concentrations recorded to remain within biologically plausible limits. The specific treatments and target concentrations (ng/g) were: clothianidin (mean = 15.28, max = 56.55), atrazine (mean = 8.59, max = 238.7), *S*-metolachlor (mean = 1.23, max = 15.31), azoxystrobin (mean = 0.67, max = 31.06), pyraclostrobin (mean = 8.51, max = 211.75), and trifloxystrobin (mean = 4.48, max = 164.25). In addition, we included a mixture treatment that included all six pesticides together at mean/max concentrations. These pesticide treatments were compared against two controls—an untreated and a solvent (acetone) application to control for the fact that pesticides were diluted in a carrier—resulting in 16 total treatments [= (6 individual pesticides × 2 concentrations) + (1 mixture × 2 concentrations) + 2 controls].

We simulated dietary exposure by applying pesticide treatments to milkweed leaves that were subsequently fed to monarch larvae (see below section); however, individuals were also likely exposed topically as they crawled on the leaf surface and, thus, we cannot truly differentiate oral from contact. This exposure route was used, rather than applying compounds directly to the exterior of the insect cuticle, because some of the pesticides are systemic and likely taken up by plant roots (e.g., clothianidin), in which case monarchs come into contact with active ingredients while feeding on contaminated leaves.

To experimentally treat monarchs at the target dose, we harvested leaves from potted, greenhouse-grown milkweeds. After cutting at the base of the petiole, we rinsed leaves in distilled water, and removed leaf cores (20 mm diameter) using a cork borer, avoiding major leaf veins. Leaf discs were temporarily stored at 4 °C before being treated and collected daily to avoid wilting or biochemical degradation.

High purity pesticides (ordered from Sigma-Aldrich or ChemService, Inc.)—clothianidin (99.0%), atrazine (98.1%), *S*-metolachlor (97.6%), azoxystrobin (99.5%), pyraclostrobin (99.9%), trifloxystrobin (99.4%)—were diluted in 1 mL of acetone as an initial stock, from which we created subsequent serial dilutions in acetone to a final volume of 15 mL. Acetone was used because the pesticides readily dissolve in it and it evaporates quickly from the leaf surface. Target pesticide concentrations (ng/g) were adapted to the average sized milkweed leaf disc (65.7 mg) and applied to the abaxial surface in 20 μL aliquots using an Eppendorf repeater pipette. Treated discs were left to dry in the fume hood for 30 min to ensure that the acetone had fully evaporated, before being used to feed monarch larvae. Discs were handled with forceps specific to each pesticide treatment to avoid cross-contamination.

The above-described methodology was also used in a related study on monarch choice, during which we used LC/MS analysis of treated leaf discs to confirm that the target concentrations were achieved. This analysis (data reported in^53^) demonstrated that all six of the pesticides tested were at levels within ca*.* 5% of the field value that applications were based on. Clothianidin mean/max treatments, for instance, were recorded at 14.36 and 54.47 ng/g, respectively, from treated leaf discs; targeted concentrations from field data were 15.28 and 56.55 ng/g.

### Monarch development

This experiment was conducted in two separate trials; the first, was from June 30 to July 17, 2017 and the second from September 11 to October 2, 2017. Each trial contained 5 biological replicates of each treatment, with a replicate being considered an individual caterpillar, reared from the neonate to adult stage (n = 180 total monarchs across trials). Experimental larvae were reared in a greenhouse on the Purdue University campus (West Lafayette, Indiana, USA); although this environment was warmer and more variable (i.e., temperature fluctuations) than laboratory growth chambers, we noticed that monarchs perform better when reared under natural light conditions.

Recently hatched larvae were randomly assigned to a pesticide treatment and weighed using a semi-micro balance, before being placed in an individually labeled 5.5 oz plastic container with a damp strip of paper for humidity and 2–3 cm incision in the lid to provide some ventilation. Containers were arranged in a randomized complete block design, with one full replicate of each concentration (i.e., 9 treatments) grouped together. The spatial arrangement of blocks within the greenhouse was also randomized and rotated several times throughout the experiment to reduce minor environmental differences due to positional effects. Containers were kept within a PVC frame covered with shade cloth to reduce direct sunlight and a fan operating at low speed to cool the area even further. Each day, we added a set of newly treated leaf discs, recorded larval survival, removed frass, replaced paper strips, and cleaned containers with water. The number of discs added per container daily increased over time as larvae grew and required more leaf tissue. Any leaf disc fragments that had not been eaten were taken out and photographed under a transparent 4 × 4 mm grid to later quantify herbivory per day per caterpillar.

Upon completion of the larval stage, pupae were weighed on an analytical balance and moved to mesh cages (95.25 × 57.15 × 59.69 cm LWD) separated by treatment. Cardboard platforms were placed at the top of cages to provide shade and a structure to attach pupae. Pupal survival and development time (i.e., no. days until adult emergence) were recorded.

When adults emerged, their date of emergence and sex were recorded. After their wings dried, but before they started flying, a small amount of paint from Painters’ opaque paint markers (Elmer’s Products, Inc.) was applied to the hindwing with a fine paintbrush to track butterflies by date of emergence and register the day of death. A different paint color was used each day to differentiate between emergence dates. Individuals that emerged the same day were marked with the same color. Butterflies were fed in cages with banana and tangerine slices, water, and Gatorade^54,55^, which were replaced every other day. To minimize temperature-induced stress (> 30 °C), cages were misted with cold water and fans were placed on nearby benches to increase airflow. Cages were checked daily and adult longevity was calculated as time from butterfly emergence from their pupae until death.

After a butterfly died, date of death was recorded and its size was measured by folding the wings together and measuring from the farthest tip of the forewing to the first white dot on the thorax^56^. There is a linear relationship between forewing length and size in monarchs^57^ and butterflies in general^58^.

### Statistical analysis

Larval survival across treatments was analyzed using Kaplan–Meier survival curves and the log-rank test to compare among treatments. A posthoc pairwise comparison between the control and each treatment was done only for maximum concentrations. Based on sample sizes, a 50% reduction in survival (5/10 mortality) would be necessary to detect a significant (*P* < 0.05) effect of individual pesticide treatments. These analyses were performed using the package survivalAnalysis^59^ in R.

A two-way ANOVA was used to test the effects of trial and pesticide treatments on the response variables: leaf area consumed (herbivory), immature development time (larval + pupal development), pupal weight, adult longevity, and adult size (wing length). Univariate ANOVAs were performed for each response variable with separate analyses conducted for the mean and maximum concentrations. Normality assumptions were verified using QQ plots and homogeneity of variances using Levene’s test. A post hoc pairwise t-test comparison was used to test differences in adult size between the control and other treatments. The Holm–Bonferroni p_adjusted method was used to control the overall Type I error. Analyses were performed in R 3.6.2 using the packages tidyverse^60^, ggpubr^61^, rstatix^62^, car^63^ and ggplot2^64^.

We also used the package ggcorrplot^65^ to analyze and graph the correlation matrix between monarch performance variables: larval development time, herbivory, pupal weight, adult size, and adult longevity. This analysis combined individuals from all treatment groups and was performed separately on monarchs reaching adulthood, reared at mean and maximum pesticide concentrations for trials 1 and 2.

Although raw data are provided in Table 1, figures were created using effect sizes^66^ to control for random trial variation by standardizing treatment data against the control in each block, using the formula: ln(treatment/control). We used the R package pairwiseCI^67^ to calculate 95% confidence intervals surrounding control vs. individual pesticide treatment comparisons to estimate uncertainty in our findings (Supplementary Table 1).

**Table 1:** Raw data showing the sample size (n), mean, and variation (SE) for monarch responses at mean (A) and maximum (B) pesticide concentrations tested.

Treatments	n	Herbivory (cm^2^)	Pesticide consumed (ng)	n	Immature developm. (# days)	n	Pupal weight (g)	n	Adult longevity (# days)	Adult wing size (cm)
		Mean + SE	Mean + SE		Mean + SE		Mean + SE		Mean + SE	Mean + SE
**(A) Mean concentrations**										
Control	10	210 + 15.2	0 + 0	9	21.9 + 1.47	9	1.41 + 0.14	8	10.1 + 1.26	4.65 + 0.08
Acetone	9	247 + 26.8	0 + 0	9	23.8 + 1.89	9	1.18 + 0.14	9	10.6 + 1.76	4.27 + 0.17
Clothianidin	9	221 + 18.5	66.4 + 5.5	8	23.6 + 1.76	8	1.14 + 0.10	8	10.5 + 1.76	4.59 + 0.12
Atrazine	8	224 + 24	45.5 + 4.9	8	22.2 + 1.56	8	1.45 + 0.15	8	17.1 + 4.13	4.44 + 0.10
*S*-Metolachlor	10	248 + 24.6	7.05 + 0.7	10	22.8 + 1.47	10	1.2 + 0.12	10	12.5 + 1.86	4.44 + 0.13
Azoxystrobin	8	227 + 24.2	2.28 + 0.2	7	23.6 + 1.99	7	1.08 + 0.13	6	15.0 + 2.71	4.07 + 0.07
Pyraclostrobin	8	234 + 26.2	57.4 + 6.4	8	22.6 + 1.66	8	1.08 + 0.13	7	16.4 + 1.76	4.26 + 0.07
Trifloxystrobin	7	207 + 27.9	26.8 + 3.6	6	19.7 + 1.67	6	1.55 + 0.19	5	21.6 + 4.98	4.08 + 0.12
Mix	7	243 + 25.6	156 + 16.4	7	24.7 + 1.51	7	1.08 + 0.18	5	13.4 + 2.73	4.44 + 0.16
**(B) Max. concentrations**										
Control	10	251 + 23	0 + 0	10	22.8 + 1.49	10	1.41 + 0.13	10	14.6 + 4.20	4.64 + 0.05
Acetone	7	206 + 20.3	0 + 0	6	22 + 1.81	6	1.55 + 0.17	5	16.2 + 4.47	4.34 + 0.30
Clothianidin	7	283 + 28.3	322 + 32.3	7	24.7 + 1.76	7	1.2 + 0.17	7	10.2 + 1.96	4.29 + 0.11
Atrazine	8	217 + 23.8	1,495 + 164	7	22.4 + 1.99	7	1.32 + 0.16	6	15.0 + 3.29	4.1 + 0.24
*S*-Metolachlor	9	230 + 21.1	75.1 + 6.88	9	22.6 + 1.67	9	1.45 + 0.18	9	14.6 + 2.27	4.44 + 0.10
Azoxystrobin	5	209 + 32.1	101 + 15.6	5	20 + 1.52	5	1.44 + 0.21	5	14.9 + 3.11	4.46 + 0.10
Pyraclostrobin	10	247 + 25.2	1,225 + 125	8	22 + 1.7	8	1.5 + 0.163	8	15.0 + 1.56	4.4 + 0.10
Trifloxystrobin	6	250 + 28.4	827 + 93.7	6	23.3 + 1.96	6	1.29 + 0.18	6	13.3 + 2.43	4.52 + 0.13
Mix	9	217 + 20.7	3,450 + 329	9	22.2 + 1.67	9	1.42 + 0.17	9	11.5 + 0.89	4.36 + 0.08

Data combine trials 1 and 2.

## Results

We started the experiment with 180 individuals (= 9 treatments × 2 concentrations × 10 biological replicates), from which 33 died as larvae, 8 died during the pupal stage, and 8 either died during adult eclosion or emerged with deformed wings and thus were removed from the analysis. This developmental mortality resulted in 73% of monarchs surviving to a fully functioning adult across all treatments.

Of those who survived to adulthood, we found significant trait correlations across life stages for several pairwise comparisons (Supplementary Fig. 1). While the presence and strength of these relationships varied among trials, the most consistent—3 out of 4 tests—were positive associations between larval development time and herbivory (i.e., larvae who lived longer, ate more; r = 0.44–0.51) and pupal weight and wing length (i.e., heavier cocoons produced adults with bigger wings; r = 0.40–0.71).

**Figure 1:**
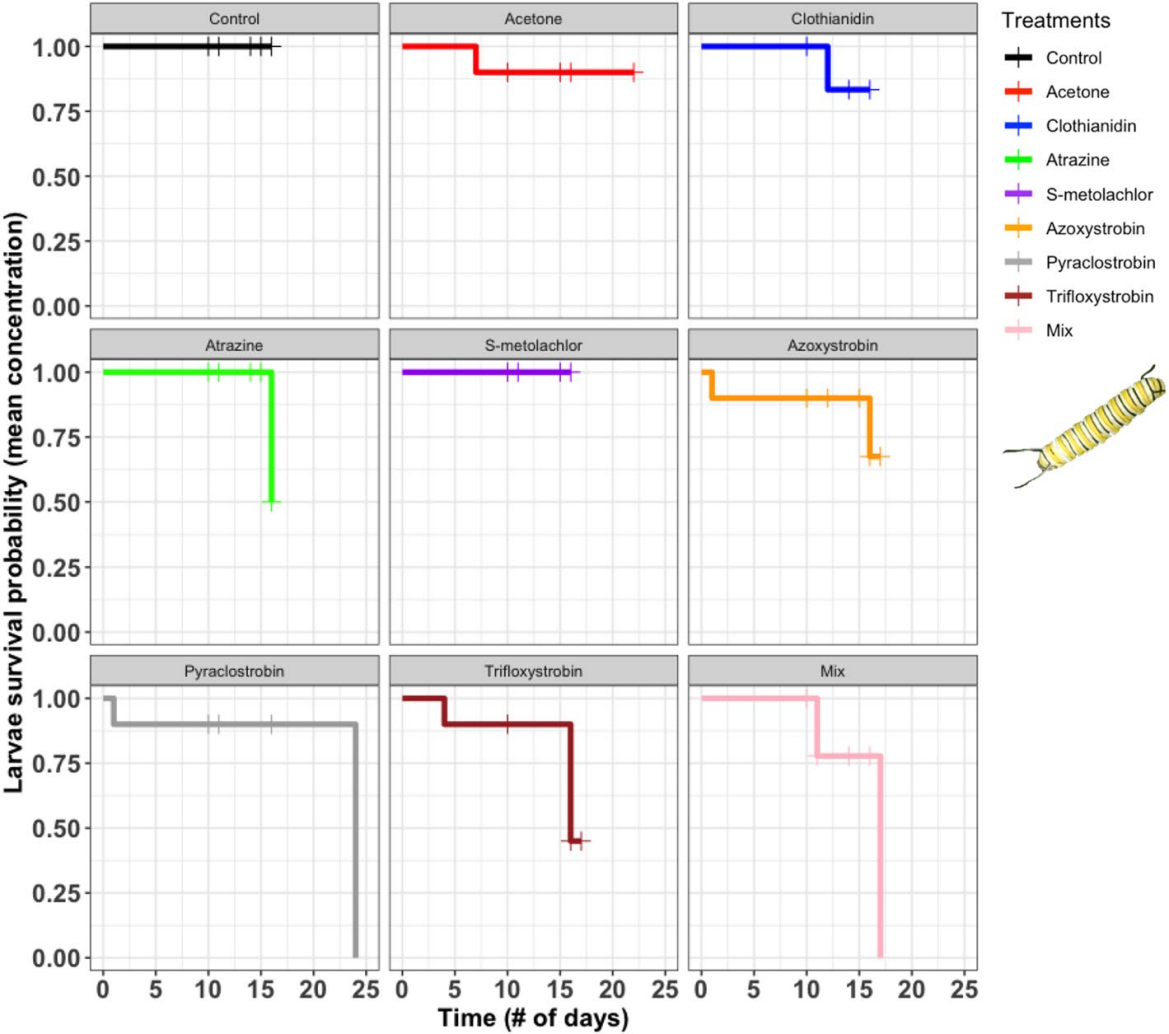
Survival curves comparing daily larval survival of monarchs in response to the untreated control, solvent control (acetone), six pesticides, and their combination (mix) at mean concentrations. Cross hatches on curves represent larvae that pupated. Photo credit: Paola Olaya-Arenas.

### Larval survival

**Figure 2:**
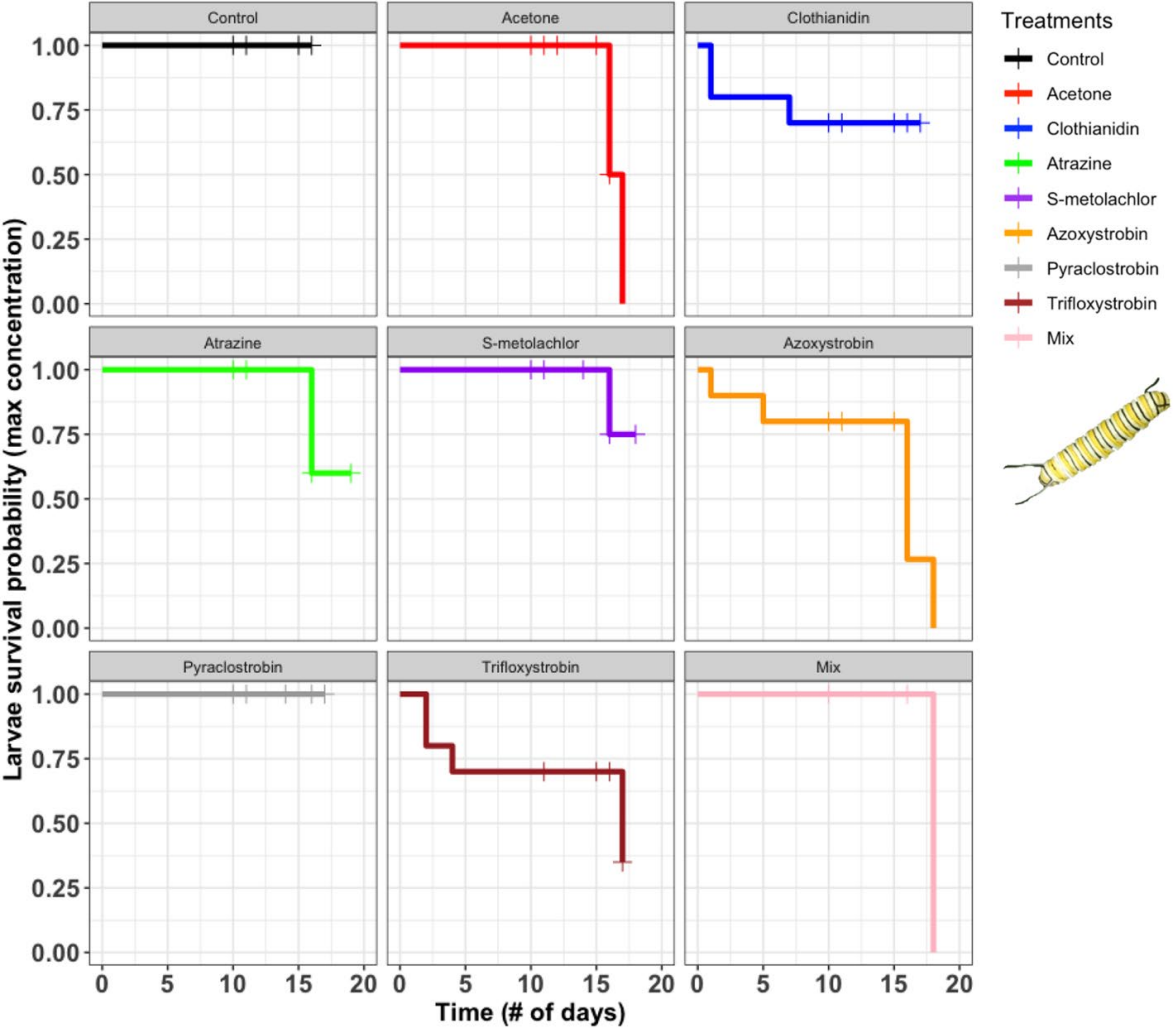
Survival curves comparing daily larval survival of monarchs in response to the untreated control, solvent control (acetone), six pesticides, and their combination (mix) at maximum concentrations. Cross hatches on curves represent larvae that pupated. Photo credit: Paola Olaya-Arenas.

None of the pesticide treatments affected the daily survival of monarch larvae when ingested at their mean concentrations (log-rank = 0.65; Fig. 1). However, pesticides at their maximum concentrations had a marginally significant impact on larval survival (log-rank = 0.064; Fig. 2). Pairwise comparisons between survival curves revealed a significant difference only between the control and azoxystrobin (50% survival; χ^2^ = 4.6, *P* = 0.03) with marginal negative effects caused by trifloxystrobin (60% survival; χ^2^ = 3.4, *P* = 0.07) and clothianidin (70% survival; χ^2^ = 3.4, *P* = 0.07). In the untreated control, 100% of larvae (20 out of 20) survived to pupation across both trials.

### Herbivory

Similar to larval survival, there was no effect of pesticides on the amount of leaf tissue consumed by monarch larvae when presented at their mean concentrations (Table 2A); however, at maximum concentrations there was a marginal (*P* = 0.06) impact on herbivory (Table 2B). Specifically, atrazine or the mixture of all pesticides combined caused a 14% reduction in leaf consumption (Supplementary Fig. 2). Trial strongly affected herbivory—but did not interact with treatment—primarily because we increased the number of leaf discs from 10 to 20 between trials 1 and 2 to ensure that food was not limiting for larvae.

**Table 2:** Results from ANOVA testing the main and interactive effects of trial and pesticide treatment on monarch development at mean (A) and maximum (B) concentrations.

Response variables	Predictor variables	df	F	*P*	ges
(**A) Mean concentrations**					
Herbivory	Trial	**1.58**	**262.17**	**0.00**	**0.82**
	Pesticide	8.58	1.60	0.15	0.18
	Trial × pesticide	8.58	1.38	0.23	0.16
Immature development	Trial	**1.54**	**1692.76**	**0.00**	**0.97**
	Pesticide	8.54	1.15	0.35	0.15
	Trial × pesticide	8.54	1.68	0.12	0.20
Pupal weight	Trial	**1.54**	**113.17**	**0.00**	**0.68**
	Pesticide	8.54	1.59	0.15	0.19
	Trial × pesticide	8.54	1.46	0.19	0.18
Adult longevity	Trial	1.49	0.00	0.96	0.00
	Pesticide	**8.49**	**2.02**	**0.06**	**0.25**
	Trial × pesticide	**7.49**	**1.93**	**0.09**	**0.22**
Wing length	Trial	**1.49**	**18.67**	**0.00**	**0.28**
	Pesticide	**8.49**	**2.78**	**0.01**	**0.31**
	Trial × pesticide	7.49	0.37	0.92	0.05
**(B) Max. concentrations**					
Herbivory	Trial	**1.53**	**595.81**	**0.00**	**0.92**
	Pesticide	**8.53**	**2.02**	**0.06**	**0.23**
	Trial × pesticide	8.53	1.02	0.43	0.13
Immature development	Trial	**1.49**	**1658.36**	**0.00**	**0.97**
	Pesticide	8. 49	0.69	0.70	0.10
	Trial × pesticide	8. 49	0.78	0.62	0.11
Pupal weight	Trial	**1.49**	**149.60**	**0.00**	**0.75**
	Pesticide	8. 49	0.61	0.76	0.09
	Trial × pesticide	8. 49	0.53	0.83	0.08
Adult longevity	Trial	1.47	0.83	0.37	0.02
	Pesticide	8.47	0.32	0.96	0.05
	Trial × pesticide	8.47	0.51	0.84	0.08
Wing length	Trial	**1.47**	**15.47**	**0.00**	**0.25**
	Pesticide	**8.47**	**1.85**	**0.09**	**0.24**
	Trial × Pesticide	8.47	0.93	0.50	0.14

The generalized eta squared (ges) calculates how much variation in the response is explained by predictor variables. Significant (*P* < 0.05) or marginally significant (*P* < 0.1) effects are bolded for emphasis.

### Development time

Most monarchs developed through their immature (larval + pupal) stage in 19–25 days (Table 1). None of the pesticide treatments at either concentration affected this developmental rate (Table 2).

### Pupal weight

As with development time, the weight of monarch pupae was unaffected by any of the pesticide treatments at both concentrations tested (Tables 1, 2).

### Adult longevity

**Figure 3:**
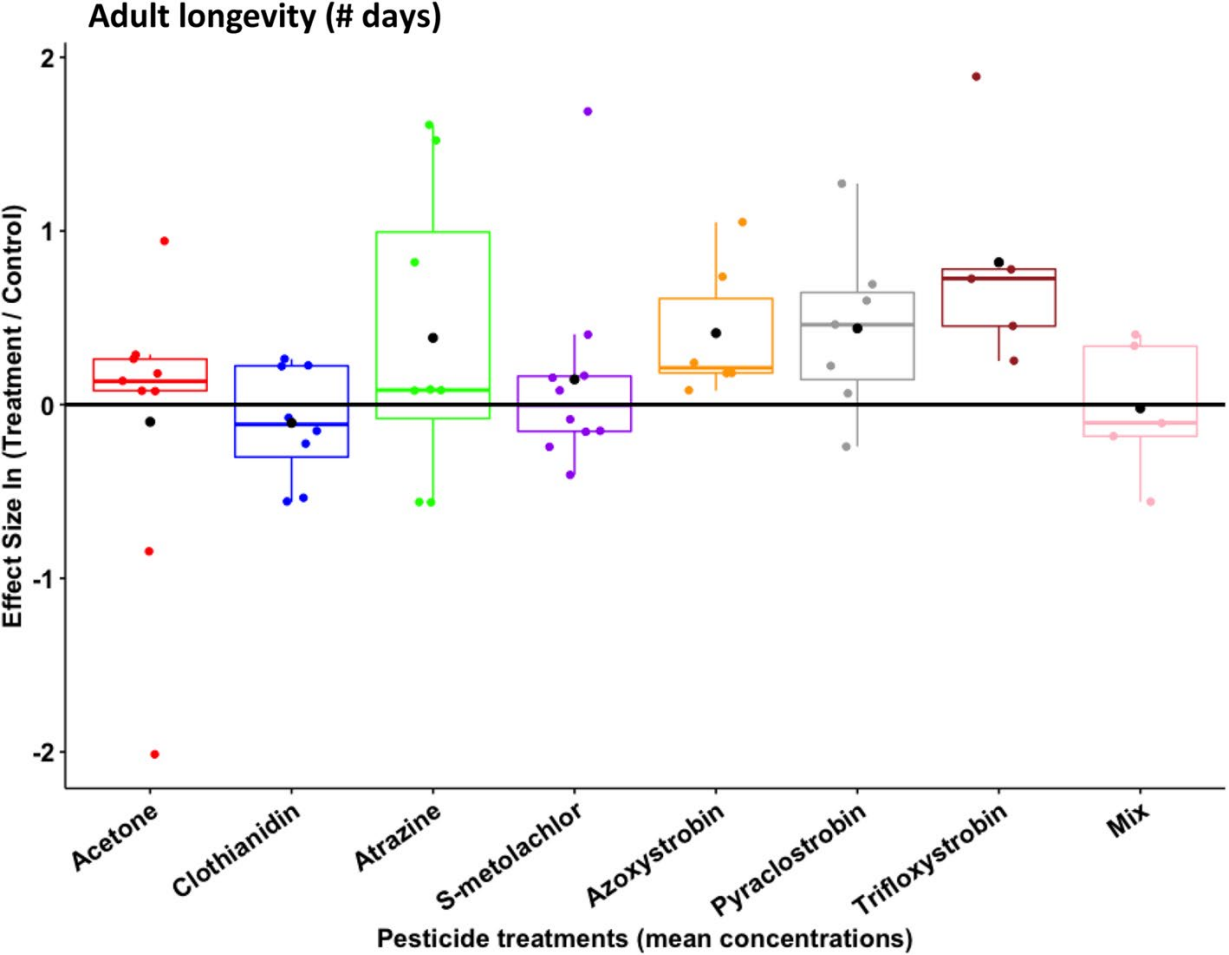
Butterfly adult longevity (# of days living), after being reared on milkweed leaves treated with the solvent control (acetone) and those experimentally treated to simulate the mean concentrations of field values for six pesticides and their combination (mix). An effect size at zero—i.e., the black horizontal line—represents no quantitative difference between the treatment and control, whereas positive or negative values indicate relative increases or decreases, respectively. Box plots show median values with 95% confidence intervals. Black circles are the treatment mean and colored circles are individual data points.

Butterflies lived in captivity for approximately 10–22 days (Table 1). Pesticide treatment had a marginally significant (*P* = 0.06) effect on adult longevity at mean concentrations (Table 2A), but there was no impact at maximum concentrations (Table 2B). Interestingly, adult longevity was extended after larvae were reared on certain pesticides at the lower concentration used; most notably, monarchs reared with exposure to the three fungicides—azoxystrobin, pyraclostrobin, trifloxystrobin—lived for 50–100% longer than control individuals (Fig. 3).

### Adult size

**Figure 4:**
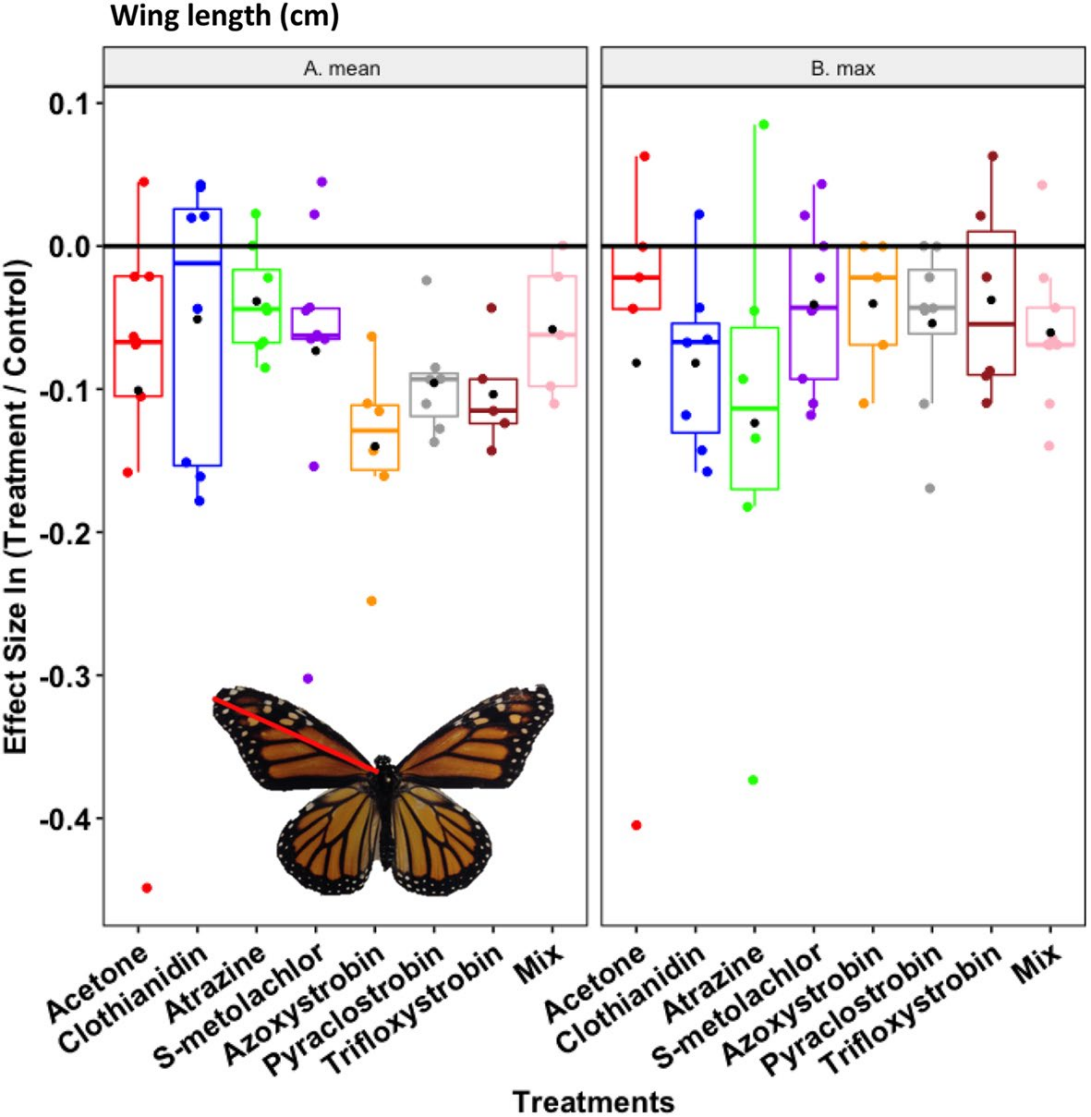
Butterfly adult size (cm wing length; red line on inset monarch photo), after being reared on milkweed leaves treated with the solvent control (acetone) and those experimentally treated to simulate the A) mean and B) maximum concentrations of field values for six pesticides and their combination (mix). An effect size at zero—i.e., the black horizontal line—represents no quantitative difference between the treatment and control, whereas positive or negative values indicate relative increases or decreases, respectively. Box plots show median values with 95% confidence intervals. Black circles are the treatment mean and colored circles are individual data points. Photo credit: Paola Olaya-Arenas.

Monarch wing length ranged from 4 to 5 cm (Table 1) and was the most consistently affected response variable with a significant pesticide treatment effect at mean concentrations (Table 2A) and marginally significant effect (*P* = 0.09) at maximum concentrations (Table 2B). In virtually all cases, wing size tended to be lower when reared as larvae with pesticide residues; however, after a Holm–Bonferroni correction, individual effects of azoxystrobin and trifloxystrobin were significant, with a 12.5% reduction in wing length compared to the control (Fig. 4A). While these fungicides also trended toward lower wing length at maximum concentrations, atrazine-reared monarchs developed to the smallest final size of all treatments (Fig. 4B).

## Discussion

Isolating and experimentally testing the impact of each hypothesized factor contributing to monarch decline is critical for establishing a robust and scientifically grounded restoration plan. Although the potential threat from dietary exposure to pesticides is widely acknowledged, our experiment represents the only life cycle evaluation to date of monarchs reared with agricultural pesticides over their complete larval development on their primary host-plant, *A. syriaca*. As outlined in the below sections, monarch responses varied depending on developmental stage, pesticide type, and concentration. Independent of these factors, however, we found little evidence for synergism or other forms of non-additivity in the mixed pesticide ‘cocktail’ treatment. This outcome stands in contrast to studies, mainly involving honey bees, that report synergistic reactions to co-exposure of fungicides and neonicotinoids, among other combinations of active ingredients (but see^68,69^). Thus, we primarily considered the main effect of individual pesticide residues rather than interactions between the different components, even though they frequently co-occur on the same plant or leaf in the field^52^.

It is also important to consider the advantages and limitations of our experimental design. To precisely simulate field values, we used leaf cores where specific pesticide quantities that match target concentrations could be applied. This level of precision comes with a few drawbacks. For example, leaf cores sever the milkweed laticifers that deliver anti-herbivore latex to feeding sites, thereby limiting the role of plant defense compared with intact leaves. Similarly, we applied pesticides to the leaf surface and therefore cannot definitively conclude whether exposure was dietary or contact. For systemic compounds, pesticides could be transported internally from the soil through the roots rather than aerially deposited on the leaf surface, although pesticide exposure routes for milkweed are unknown. A recent study^47^ applied clothianidin to the soil of potted milkweed plants to effectively generate a wide spectrum of leaf concentrations, demonstrating that this approach is methodologically feasible for certain compounds.

### The role of neonicotinoids in monarch decline

Based largely on an earlier study^46^ reporting sublethal effects of clothianidin on monarch larvae at levels as low as 1 ng/g with an LC_50_ of 15.63 ng/g, we expected to find strong deleterious effects of this insecticide on survival and development at the two concentrations tested (15.28 and 56.55 ng/g). This was especially the case since prior work used a short-term (36 h) pulsed bioassay, whereas we employed a longer-term continuous exposure, which should result in a correspondingly stronger impact. However, clothianidin had relatively weak effects at both concentrations for all variables measured, with a moderate and only marginally significant reduction in survival at the maximum concentration tested. While our statistical power to tease apart relatively minor differences in mortality was low, this outcome corresponds closely with data reported by Bargar et al.^47^ who calculated clothianidin LC_50_ values ranging from 47 to 205 ng/g for monarchs experiencing chronic exposure over their entire larval period, and Krishnan et al.^45^ who reported a range of 800–7,800 ng/g for acute exposure across instars. Given that 56.55 ng/g was the maximum value we ever recorded in > 1,000 milkweed leaf samples at multiple sites over 2 years^52^ and other studies reported even lower maximum concentrations (4.02 ng/g in field samples in South Dakota^46^), we consider it unlikely that clothianidin is a major driver of monarch declines.

One possible reason for their physiological tolerance of clothianidin is that monarchs are selected for enhanced detoxification of xenobiotics due to evolving as a specialist on a highly toxic host-plant^70^. Milkweeds are rich in cardenolides and other secondary metabolites, placing strong selective pressure on their herbivores^71,72^. This co-evolutionary process could make them predisposed to detoxifying pesticides; indeed, the pre-adaptation hypothesis predicts that host-plant chemical defenses prime an insect lineage to evolve insecticide resistance, a pattern supported in a review of published data^73^. It remains unclear whether cardenolide tolerance confers cross-resistance to clothianidin. However, a direct comparison between LD_50_ values concluded that specialist monarchs are less sensitive than generalist honey bees for most insecticide modes of action evaluated, including neonicotinoids^45^. Thus, using honey bees as a proxy for how all pollinators, and invertebrates in general, respond to clothianidin is probably misguided. Larval survival for another oligophagous butterfly, *Polyommatus icarus* (Lep.: Lycaenidae), was also robust to wide variation in foliar clothianidin levels (5–500 ng/g) on wild, leguminous host-plants^74^. These data suggest that clothianidin tolerance is not unique to monarchs but could be a trait that is phylogenetically conserved in specialized Lepidoptera.

Beyond their physiological response, our previously collected field data further indicate that monarch exposure to clothianidin is unlikely based on phenological asynchrony between pesticide and caterpillar^52^. We mainly detected clothianidin in leaves in June, before monarchs widely colonize milkweeds in our area. Yet, it is important to note that in the second year of the study we detected far more thiamethoxam on milkweed throughout the summer. Thiamethoxam is a biochemical precursor of clothianidin and widely used as a neonicotinoid seed treatment in the Midwestern US region. Although we did not directly test this compound, its toxicity to monarchs is comparable to clothianidin and several orders of magnitude less than more potent insecticides like beta‐cyfluthrin (pyrethroid) and chlorantraniliprole (anthranilic diamide)^45^. A recent field survey of pesticide contamination in western US milkweeds similarly concluded that chlorantraniliprole was a far greater threat to monarchs than neonicotinoids^75^.

As a caveat, the above conclusions are specific to wild milkweeds growing in agricultural areas. Neonicotinoids on milkweed in urban gardens can reach excessively high levels when treated as ornamentals, resulting in 100% monarch larval mortality^44^. Also, our assessment is specific to dietary exposure via leaf herbivory. A recent study^30^ found that adult monarch survival was lower when ingesting imidacloprid (23.5 ng/g) in their nectar diet; however, this outcome stands in contrast with Krischik et al.^44^ who reported no impact of imidacloprid at similar or higher concentrations on monarch adult survival and fecundity.

### Delayed effects of larval pesticide exposure on adult performance and potential consequences for migration

We expected the immediate impact of pesticides on larval survival and growth rate to be stronger than effects expressed on later life stages. Yet, the most consistent responses in the dataset were observed in adults with longevity and wing length. This outcome is not without precedent. The butterfly *Pieris brassicae* was reared on cabbage plants varying in the neonicotinoid imidacloprid (0–200 ppb) with a similar outcome; larval digestion and behavior were unaffected, but adult wing size was reduced^34^. As with the current study (see Fig. 4A vs. B), this prior work also found that negative effects on adult traits were relatively insensitive to wide variation in concentration beyond the untreated control. Other studies testing monarch reaction to pesticides similarly show poor correspondence between dose and response^46,47^, even though a linear relationship is often assumed. The range of values chosen in our study, while seemingly high (i.e., the max. treatments were ca*.* 4- to 50-times higher than the mean treatments), were far less than many ecotoxicology studies that vary dose by 3–4 orders of magnitude and still do not detect a clear linear pattern. However, it is also challenging to interpret the effects of dose, particularly for sublethal responses, when data are only collected on survivors and higher concentrations reduce survival.

Importantly, independent of dose, we found consistent reductions in adult wing size when larvae were reared on certain pesticide treatments. Assuming that wing size is positively correlated with migratory ability, which it appears to be in monarchs^76–79^, these data imply that delayed effects of oral exposure as larvae could be linked with successful fall migration back to overwintering grounds in Mexico. Indeed, this possibility was suggested by Inamine et al.^80^ who speculated that: “*the condition of fall migrants might be affected by the environments they experience early in life, including milkweed shortage, insecticides, or other changes in habitat quality*”. To our knowledge, ours is the first study to substantiate this possibility, showing that oral pesticide exposure as a caterpillar leads to smaller monarch butterflies. It is critically important for future studies to take this result one step further and assess migratory behavior using flight mills or related techniques to connect pesticide ingestion, wing size, and flight capability.

One particularly interesting, but counterintuitive, finding was that monarchs lived *longer* when exposed to low levels of fungicides and this benefit was consistent across the three strobilurin compounds tested. While we do not have mechanistic data to support this hypothesis, we speculate that it could be a result of microbial interactions between monarchs and their environment. Our personal observations from initial monarch rearing in the laboratory indicate that this species is highly sensitive to microbial pathogens. In fact, most monarchs did not survive larval development unless their containers were sterilized daily. It should also be noted that adults were checked for the protozoan parasite *Ophryocystis elektroscirrha* (OE), which was never observed. We suspect that fungicides on the surface of milkweed leaves could be shifting the microbiome to a community that ultimately benefits monarchs in some fashion. This potential mechanism should be investigated in the future. It is unclear if microbial influences are unique to a laboratory environment; wild monarchs may be exposed to fewer pathogenic microorganisms due to UV exposure or other factors that reduce pathogen pressure in the field.

While adult performance metrics such as size and longevity were separately considered, co-variation in these traits could lead to life history syndromes that collectively shape the ecology of monarch butterflies. For example, fungicide exposure resulted in smaller individuals that lived longer; indeed, there was some evidence for a negative correlation between wing length and adult longevity (Supplementary Fig. 1). Since higher values of body size and lifespan are both assumed to confer a fitness benefit in insects^81,82^, presumably either life history combination could be successful, depending on factors such as the seasonal generation (e.g., migratory vs. resident), landscape context (e.g., density and distribution of milkweed patches), or degree of mate competition. It should also be noted that, while adult size is generally accepted as an accurate biological reflection of the quality of the larval growth environment, numerous factors impact adult longevity^83^. We tried to control for as many of these variables as possible, but we suspect that the duration of butterfly survival in captivity is at least partly determined by environmental features like mating status, density, sex ratio, cage area, and nectar diet. Without egg data, we cannot extend these tradeoffs to fecundity; however, female size is positively correlated with egg number in monarchs^84^. The only other two correlated life history traits in our dataset were: herbivory was positively associated with larval development time and pupal weight was positively associated with wing length. Both relationships are expected, given what is known about lepidopteran growth and development across life stages.

## Conclusions

Overall, our data point to several key messages. First, based on the results from this experiment combined with several other recently published studies^45,47,52,75^, it seems unlikely that neonicotinoids are one of the primary drivers of monarch declines. This is not to say that neonicotinoids are completely innocuous, but compared with other ongoing threats (e.g., loss of milkweed host-plants and overwintering habitat), they likely play a secondary role. A continental-scale analysis that statistically teased apart the relative importance of multiple anthropogenic factors implicated in the decline ultimately came to the same conclusion^40^. However, more data are clearly needed on responses by adult butterflies to variation in exposure via nectar, understudied variables like fecundity, and sublethal effects on ecologically-relevant behaviors (e.g., mating, oviposition, migration). This conclusion has no bearing on the well-documented threat that neonicotinoids pose to bees and other invertebrates^85^.

While neonicotinoids have received the lion’s share of attention, field surveys of pesticides residues on milkweed reveal a diversity of active ingredients that warrant additional toxicity testing for monarchs^52,75^. Our data specifically point to fungicides as a group that should be investigated more in the future. Last, it is essential to consider the delayed, sublethal effects that are expressed on adult performance traits. Unfortunately, these are the most time consuming and challenging to measure but most directly linked with monarch fitness and migratory success. Pesticide risk assessment needs to consider these effects on adult parameters, particularly for migratory species, whenever possible.

## Supplementary information

Supplementary Information.

## Data Availability

All data from this study are publicly available via the Purdue University Research Repository (PURR) at: Olaya-Arenas et al. Olaya-Arenas, P. et al. (2020), “Datasets describing monarch immature and adult performance when reared on milkweed host-plants varying in pesticides” (DOI: 10.4231/K9FH-7053).
