# Supplementary material for: Larval pesticide exposure impacts monarch butterfly performance
Source: Sci Rep. 2020 Sep 2;10:14490. doi: 10.1038/s41598-020-71211-7 (PMC7468139; doi:10.1038/s41598-020-71211-7)

**Larval pesticide exposure impacts monarch butterfly performance**

Paola Olaya-Arenas^1,*^, Kayleigh Hauri^1^, Michael E. Scharf^1^ and Ian Kaplan^1^

1. Department of Entomology, Purdue University, West Lafayette, Indiana, 47907

* Corresponding author: polaya16@yahoo.com

**Supplementary Table 1.** Estimate and 95% confidence intervals for each pairwise comparison of pesticide treatments relative to the control for (A) mean and (B) maximum concentrations.

**Supplementary Figure 1.** Heat maps showing correlation among monarch performance traits across life stages. Only individuals that survived to adulthood were included. White boxes = n.s.; colored boxes show significant (P<0.05) effects with darker colors indicating a stronger correlation.


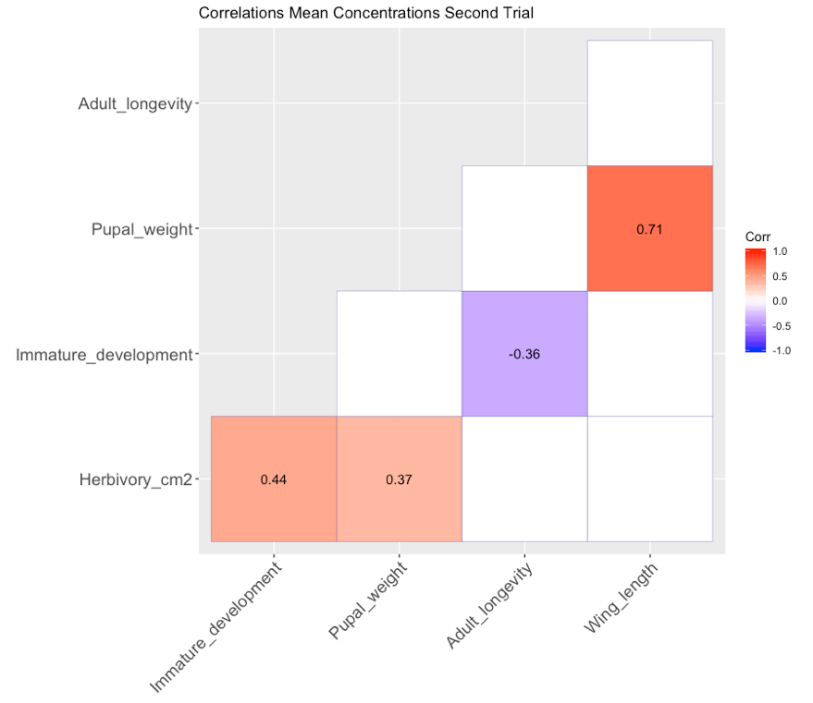

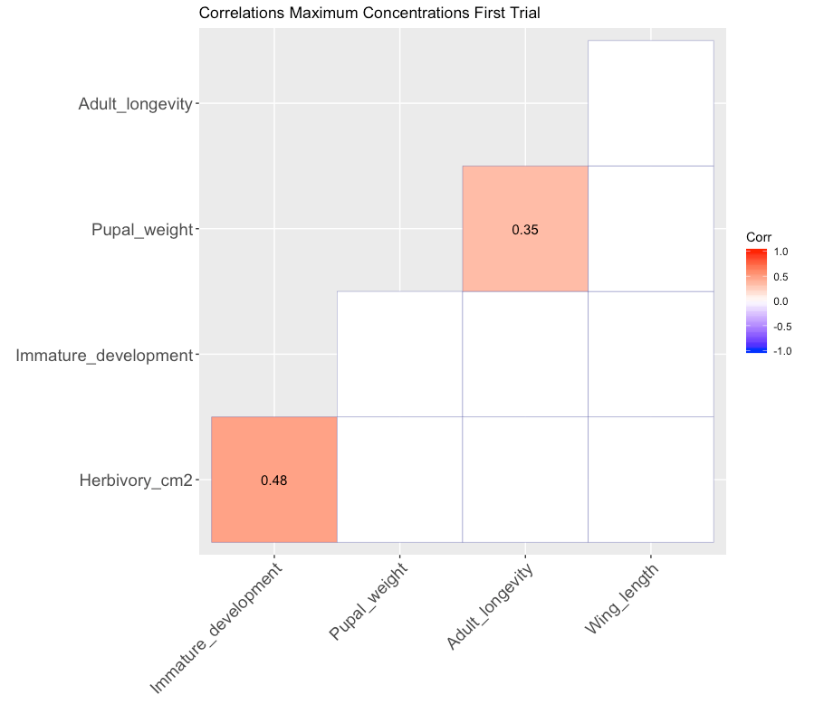

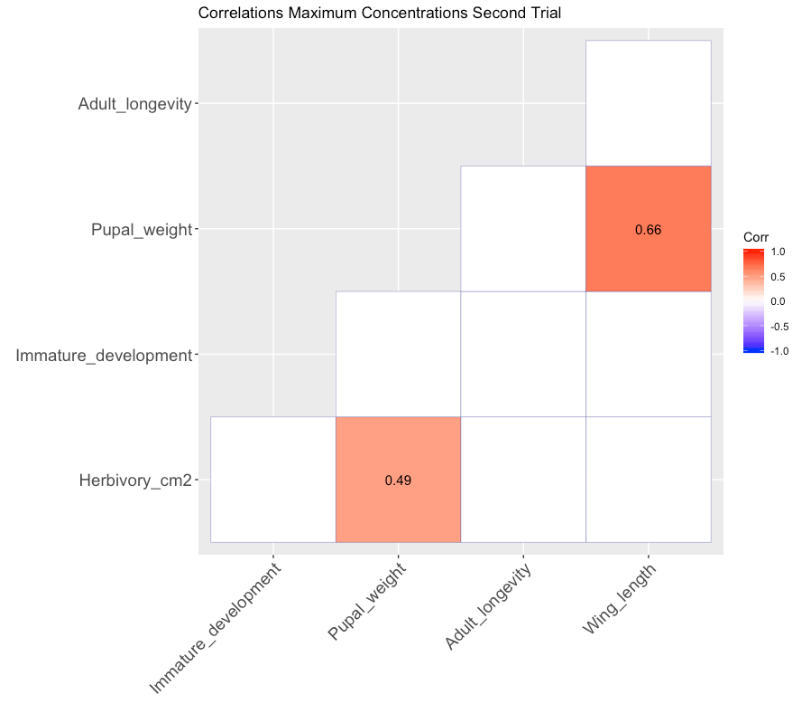

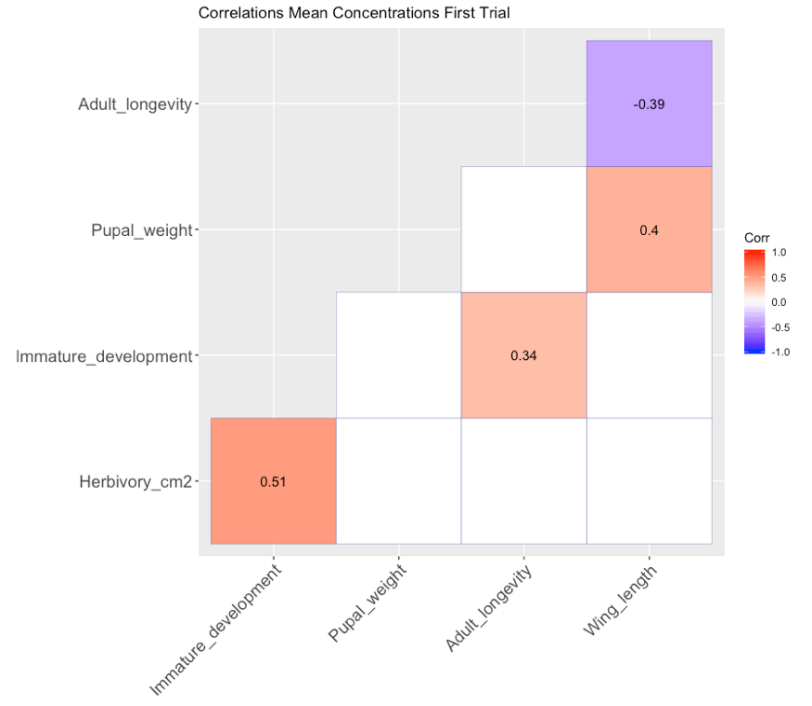


**Supplementary Figure 2.** Larval herbivory (leaf area consumed, cm^2^) while being reared on milkweed leaves treated with the solvent control (acetone) and those experimentally treated to simulate the maximum concentrations of field values for six pesticides and their combination (mix). An effect size at zero—i.e., the yellow horizontal line—represents no quantitative difference between the treatment and control, whereas positive or negative values indicate relative increases or decreases, respectively. Box plots show median values with 95% confidence intervals. Red dots are the treatment mean and black dots are individual data points. Photo credit: Paola Olaya-Arenas.


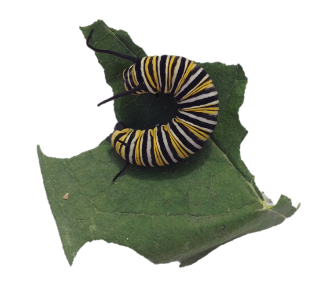

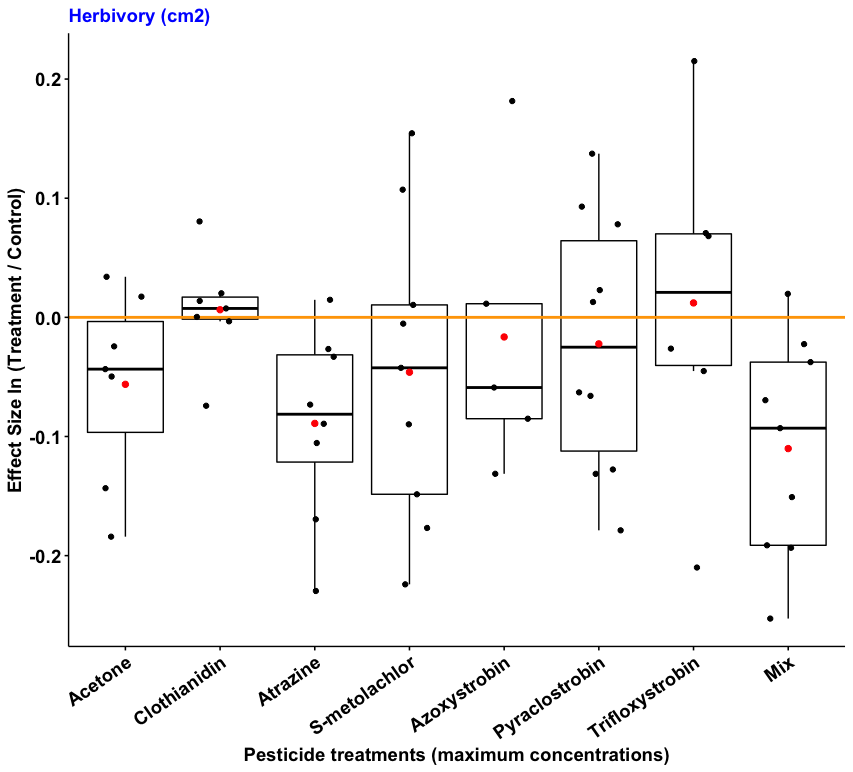

Supplement: Supplementary file 1 — Supplementary Information. [file 41598_2020_71211_MOESM1_ESM.docx]
